# Effectiveness of Denture Cleansers on Candida albicans Biofilm on Conventionally Fabricated, Computer-Aided Design/Computer-Aided Manufacturing-Milled, and Rapid-Prototyped Denture Base Resins: An In Vitro Study

**DOI:** 10.7759/cureus.63290

**Published:** 2024-06-27

**Authors:** Urvi R Echhpal, Khushali K Shah, Nabeel Ahmed

**Affiliations:** 1 Prosthodontics, Saveetha Dental College and Hospitals, Saveetha Institute of Medical and Technical Sciences, Saveetha University, Chennai, IND

**Keywords:** denture stomatitis, biofilm, candida, denture cleanser, denture base resin

## Abstract

Introduction: Conventionally fabricated denture base resins have been used for over 150 years. Newer denture base resins can provide a superior fit and may be customized to the patient's characteristics, but the literature on their cleansibility remains limited. The oral cavity can be a hub for thousands of microflora. The maintenance of complete dentures by edentulous patients depends not only on the maintenance of the patient but also on the material used, biofilm adherence, and polishability.

Materials and methods: Cuboid specimens of 10 × 5 × 2 mm were designed using the Meshmixer version 3.5 software (Meshmixer, Australia). The standard tessellation (STL) file was imported and sent for printing (NextDent, Netherlands) (Group 1), milling in polymethyl methacrylate (PMMA) (Ivotion, Ivoclar, Schaan, Liechtenstein) (Group 2), and wax milling (Upcera, China), followed by flasking, counter flasking, and packing using heat-cured acrylic resin (DPI, India) (Group 3). The obtained specimens were polished using pumice and sterilized using a UV sterilization unit. The specimens were then immersed in a suspension of candida broth. After three days of biofilm formation, a colony count was performed and noted as colony-forming units per milliliter (CFU/mL). Specimens were treated using Secure denture cleansing tablets (Ghent, New York), table salt (iodized table salt, Tata, India), Clinsodent (ICPA, Mumbai, India), and Polident denture cleansing powder (Polident, Ontario, Canada). A colony count was done after treatment, and the data were tabulated. Statistical analysis was done using SPSS software to compare the efficiency of denture cleansers in all three groups, and statistical significance was set at 0.05. The Kolmogorov-Smirnov test was done to confirm the normality of the data, followed by a one-way analysis of variance (ANOVA) test to compare the efficiency of denture cleansers on the removal of candida colonies.

Results: Milled denture base resins showed a significantly lower colony count when compared to printed and conventionally fabricated denture base resins. The denture cleansers showed high efficacy in all groups, with the most significant being Secure, which showed a mean difference ranging from 8.114 to 9.887 CFU/mL, followed by Clinsodent, showing a mean of 6.699-9.863 CFU/mL, followed closely by Polident, showing 4.964-7.114 CFU/mL, followed by table salt, being 5.254-8.920 CFU/mL. The 95% confidence interval confirmed statistical significance.

Conclusion: The highest candida colony count was demonstrated by the conventional, followed by rapid prototyping, and was least with milled denture base resins. Following treatment with denture cleansers, Secure demonstrated almost complete eradication of colonies, making it the most effective option. Salt exhibited the lowest efficiency, followed closely by Polident and Clinsodent, and the most effective was Secure denture cleanser.

## Introduction

All change brings opportunity. The introduction of a prosthesis delivered to the oral cavity creates the potential for an opportunistic infection of *Candida albicans*, often referred to as "thrush." Dentures usually elevate the accumulation of food debris and plaque formation, which, if not maintained well, can lead to ulcers, sores, and eventually denture stomatitis [1].

Epidemiological studies indicate that the prevalence of denture stomatitis among denture wearers varies widely, ranging from 15% to over 70%. The variation in prevalence rates is influenced by the diverse population samples studied. It is the single most common complication associated with denture use [2]. The mucosal surfaces in contact with dentures and the back of the tongue are common sites for denture stomatitis, characterized by erythema, swelling of the palatal mucosa, and edema in the affected areas [3]. The prevention of oral candidiasis can be ensured by religious hygiene maintenance protocols. Although the efficacy of commercially available denture cleansers on heat-cured acrylics has been demonstrated in the literature, the efficacy of newer digitally fabricated denture base resins has not been assessed.

As we witness the revolution of digital dentistry, we must note that most laboratories and hospital setups continue to fabricate denture base resins conventionally [4]. Polymethyl methacrylate (PMMA) has been considered the gold standard for removable prosthesis fabrication for many years, and it holds advantages such as low cost, acceptable esthetics, and non-toxicity. However, its successful use depends on the handling and technician skill due to the risk of surface voids and its high monomer content. These issues can result in porosities, which in turn provide a breeding ground for microbes [5]. The surface roughness and polishability of denture base resins are key factors in microbial adhesion and must be considered [6].

Denture cleaning protocols that have been commonly used apart from commercially available denture cleansers are 1% sodium hypochlorite, 2% chlorhexidine digluconate, and alkaline peroxide, which have been tested both in vivo and in vitro to assess antimicrobial effectiveness on conventionally fabricated denture base materials [7]. As multiple commercially available denture cleansers are available, their efficiency on newly developed materials must be assessed [8].

This study aimed to assess the efficacy of denture cleansers on milled, printed, and conventionally fabricated denture base resins. The null hypothesis states that there is no difference in the efficacy of denture cleansers depending on the fabrication techniques.

## Materials and methods

Study setting

**Figure 1:**
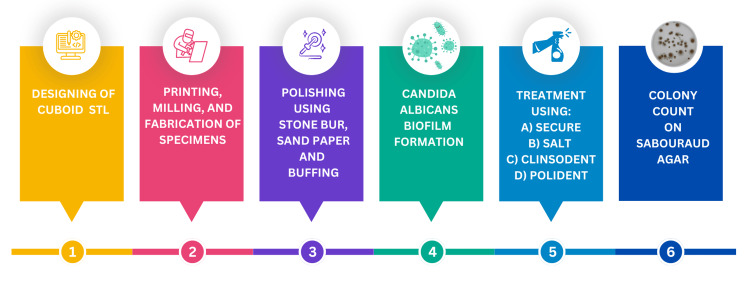
Workflow of the study

This study was conducted at a research facility in Chennai, India. Clearance for conducting this research was obtained from the university's review board (SRB/SDC/PROSTHO-2106/24/020). The workflow of the study is depicted in Figure 1.

Sample size estimation

The sample size for this in vitro study was determined using G*Power software version 3.1.2 (Heinrich Heine University Düsseldorf, Düsseldorf, Germany). The mean and standard deviation were referred to in a study conducted by Porwal et al. with p<0.50, a 5% significance level, and a study power of 0.95 [9]. This calculation resulted in a required sample size of 10.

Obtaining the test groups

**Figure 2:**
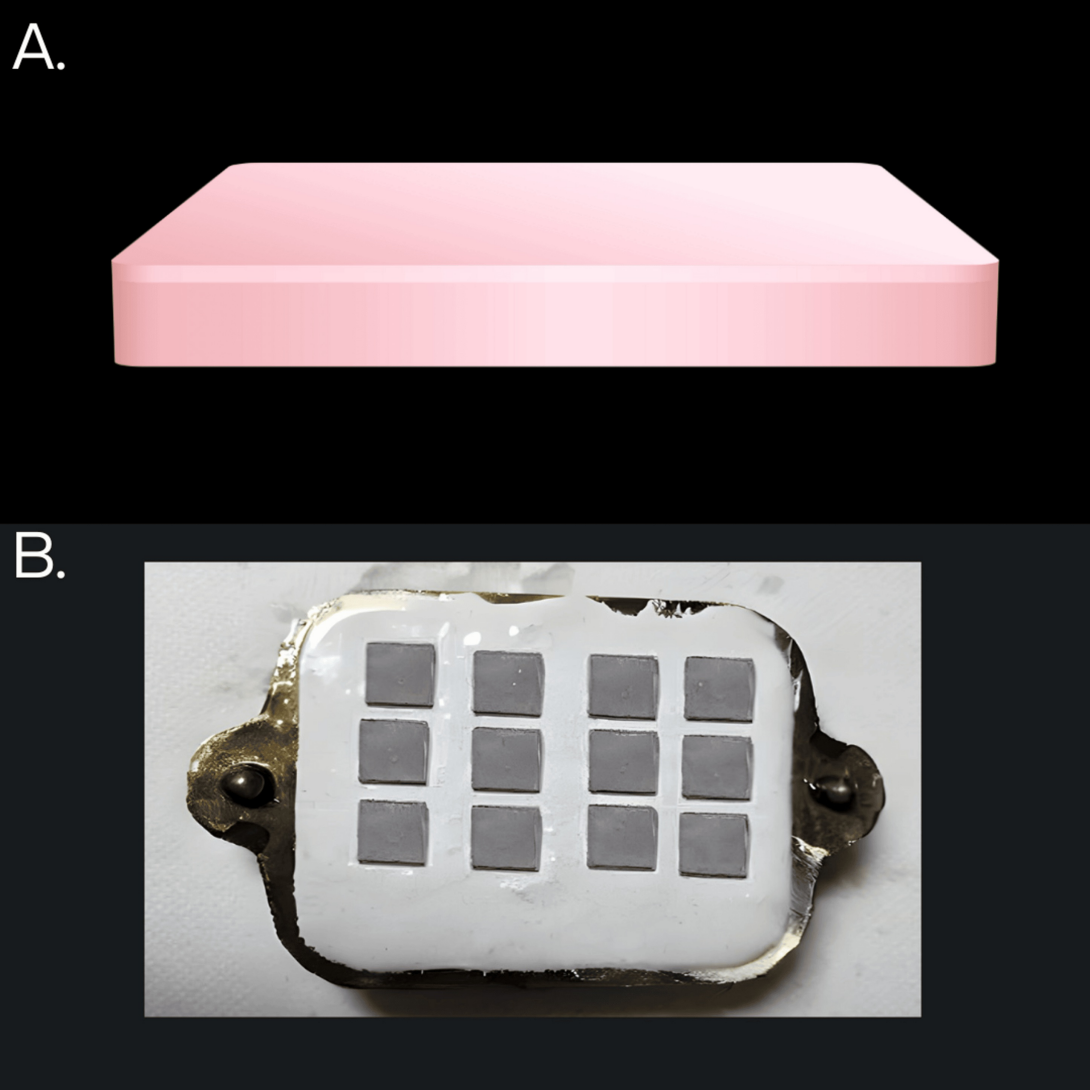
Fabrication of the specimen: (A) STL of the specimen; (B) flasking of wax specimens for a conventional denture base STL: standard tessellation

**Figure 3:**
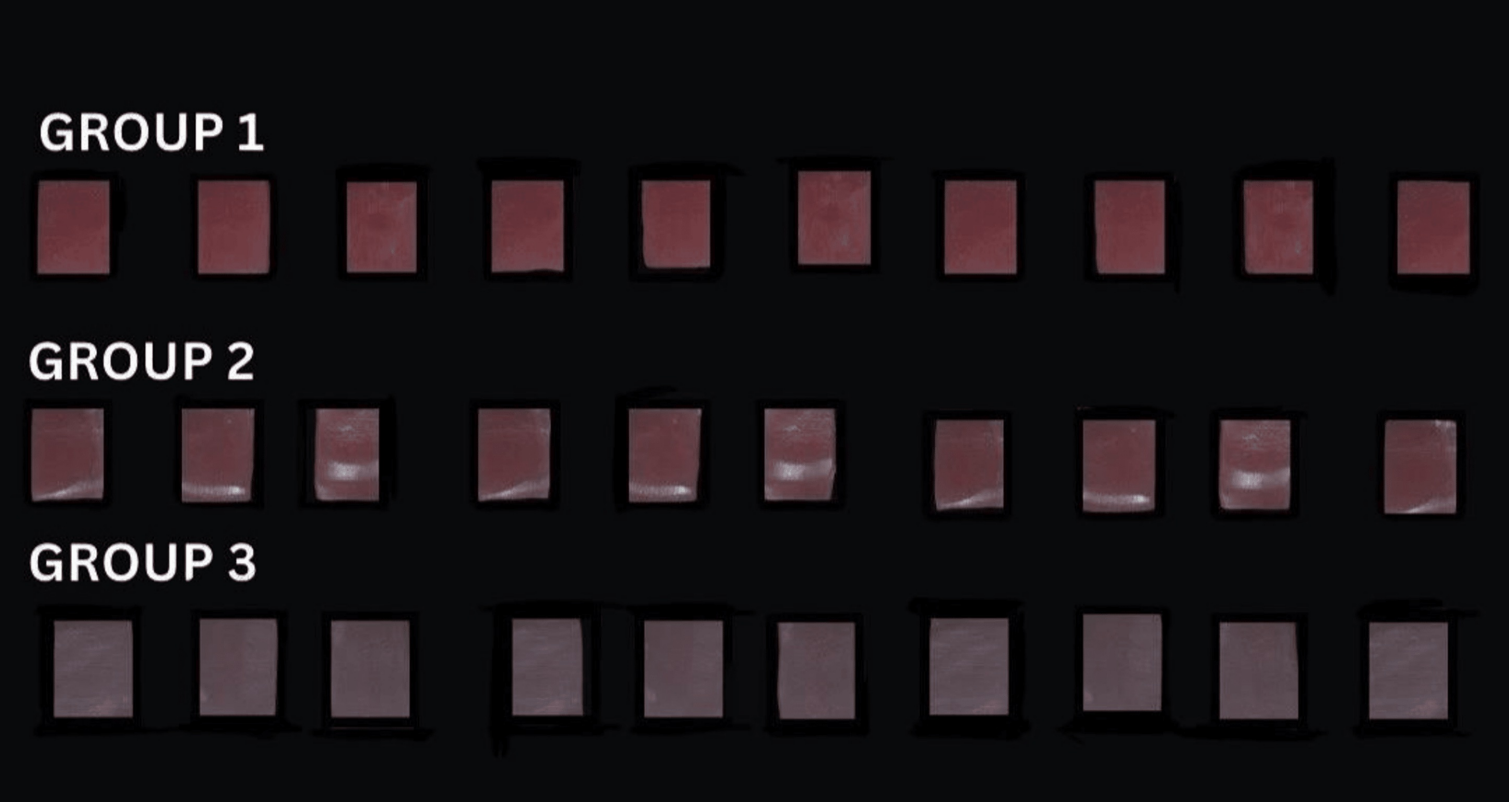
Polished specimens. Group 1: milled resin; Group 2: conventional resins; Group 3: printed resin

A cuboid was designed using the Meshmixer version 3.5 software (Meshmixer, Australia), with a length of 10 mm, width of 5 mm, and thickness of 2 mm [10]. The standard tessellation (STL) file was exported (Figure 2A). An Ivotion blank (Ivoclar, Schaan, Liechtenstein) was used for milling on a dry milling machine (IMES ICORE, CORiTECR 350i series, Hesse, Germany). Printing was done using a NextDent printer and denture base resin (NextDent, Netherlands). Wax milling was done using a dry (IMES ICORE, CORiTECR 350i series, Hesse, Germany) milling machine, after which flasking, counter flasking, dewaxing, and packing were carried out (Figure 2B) (Table 1). The samples were cleaned and dried (Figure 3). For the polishing of specimens, a coarse-grain cylindrical rubber top bur for acrylic resin (Acrylic Polisher, NMD, India) was used, followed by a fine-grain cylindrical rubber top bur (Acrylic Polisher, NMD, India). A dry polisher with a polishing cake was used.

**Table 1:** Materials used for the fabrication of the specimens

Product Name	Brand, Location	Composition
Denture 3D+	NextDent, Netherlands	Ethoxylated bisphenol A dimethacrylate;7,7,9(or 7,9,9)-trimethyl-4,13-dioxo-3,14-dioxa-5,12-diaz hexadecane-1,16-diyl bis methacrylate, 2-hydroxyethyl methacrylate, silicon dioxide, diphenyl(2,4,6 trimethyl benzoyl) phosphine oxide, titanium dioxide
Ivotion	Ivoclar, Liechtenstein	Polymethyl methacrylate
Wax blank	Upcera, China	Special paraffin wax with additives
Heat-cured	DPI, India	Polymethyl methacrylate, methyl methacrylate

Sterilization

UV sterilization was performed for 10 minutes on each side of the specimen using a sterilizer with laminar air flow (GR Scientific Instruments, Chennai, India).

Preparation of the culture media

**Figure 4:**
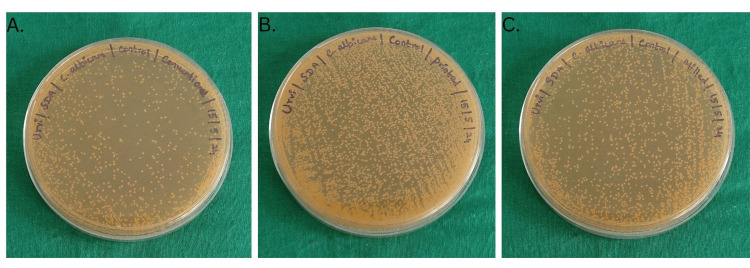
Sabouraud agar culture plates showing colony count after biofilm formation: (A) control group for conventional denture base resins; (B) control group for printed denture base resins; (C) control group for milled denture base resins

The sterilized specimens were immersed in a sterile Sabouruad Dextrose broth along with a fresh suspension of *Candida albicans*. These samples were then incubated at 37 ºC for five days to facilitate biofilm formation. Following incubation, the samples were gently agitated, swabs were collected, and lawn cultures were prepared on sterile Sabouraud Dextrose agar plates. The plates were further incubated at 37 ºC for 24-48 hours. Subsequently, the colonies were counted and recorded as colony-forming units per mL (CFU/mL) (Figure 4).

Treatment using denture cleansers

**Figure 5:**
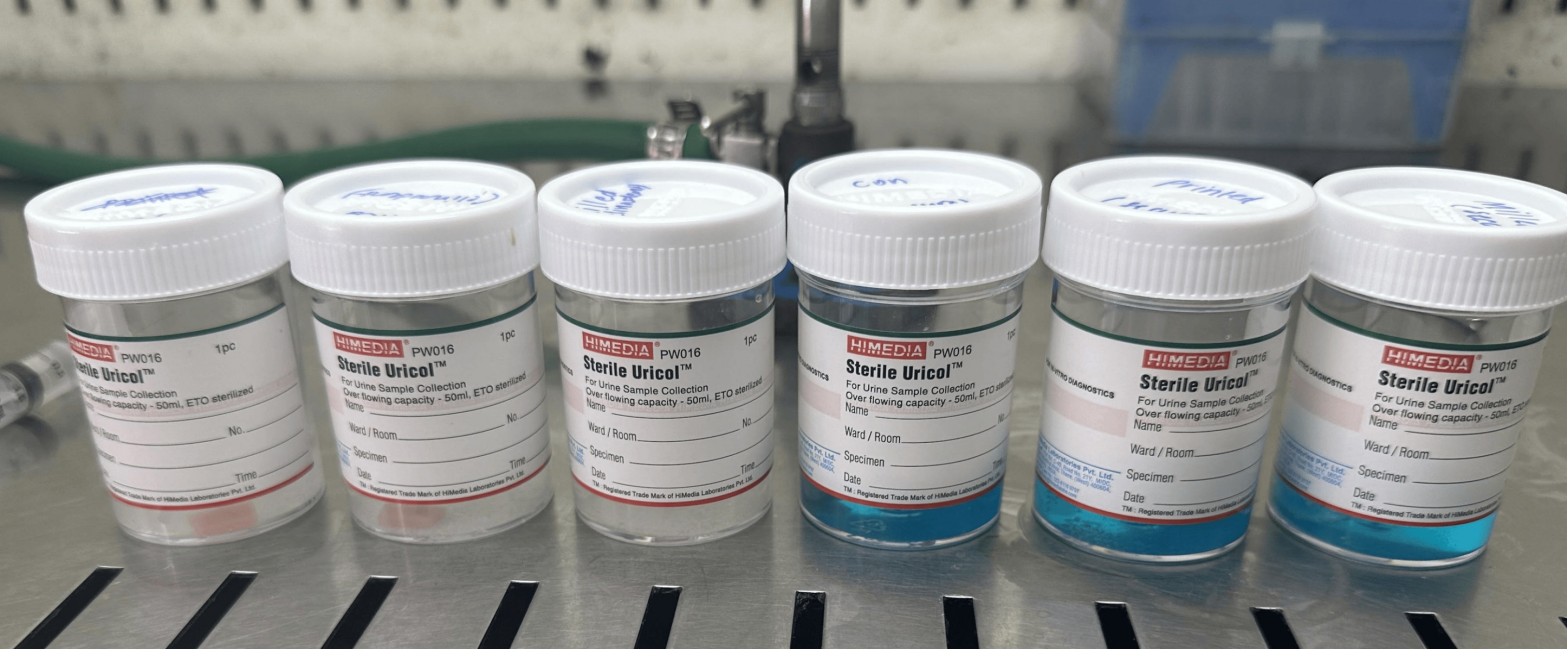
Treatment of specimens using denture cleansers

Commercially available denture cleansers, routinely used by patients in India, were used for the purpose of this study. The search was done using the online application Amazon, and the filter was set to sort products using the average customer reviews filter from the highest to the lowest. The top two products were found to be Secure denture cleaning tablets and Polident denture cleaning powder. Salt was chosen as an antimicrobial agent due to its popularity among the Indian population. A local pharmacy owner was interviewed for popular denture cleansers routinely bought by patients, and Clinsodent denture cleaning powder was recommended. Secure denture cleansing tablet (Secure, Ghent, New York), table salt (iodized table salt, Tata, India), Polident denture cleansing powder (Polident, Ontario, Canada), and Clinsodent (ICPA, Mumbai, India) (Table 2). They were sourced from the online portal, Amazon, and a local pharmacy in Chennai and mixed with saline in sterile uricols (Figure 5).

**Table 2:** Denture cleansers used, brand name, and their composition

Product Name	Brand, Location	Composition
Secure	Fittydent, India	Oxane, sodium perborate monohydrate, citric acid, sodium carbonate, sodium tripolyphosphate, sodium sulfate, and sodium lauryl sulfate
Salt	Tata, India	Edible common salt, potassium chloride, potassium iodate, and an anticaking agent
Polident	GlaxoSmithKline Plc (GSK)	Sodium perborate monohydrate, citric acid, sodium carbonate, sodium tripolyphosphate, sodium sulfate, and sodium lauryl sulfate
Clinsodent	ICPA, India	Potassium persulfate, sodium perborate, sodium carbonate, sodium sulfate, trisodium phosphate, sulfamic acid, tetrapotassium pyrophosphate, EDTA, sodium lauryl sulfate, and peppermint powder

Colony count after treatment

**Figure 6:**
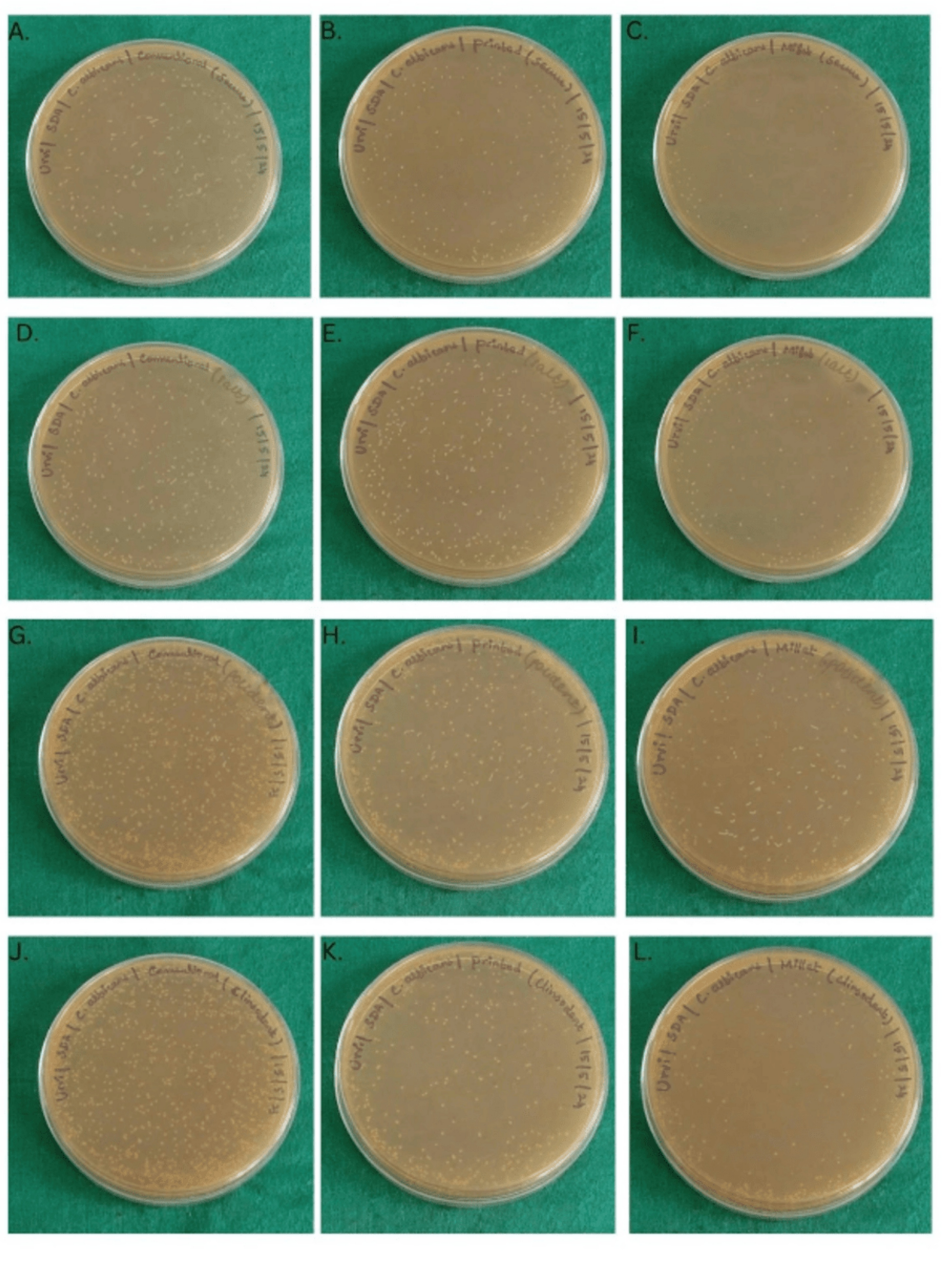
Colony count on Sabouraud agar (A) Conventional denture base resin treated with Secure; (B) printed denture base resin treated with Secure; (C) milled denture base resin treated with Secure; (D) conventional denture base resin treated with salt water; (E) printed denture base resin treated with salt; (F) milled denture base resin treated with salt; (G) conventional denture base resin treated with Polident; (H) printed denture base resin treated with Polident; (I) milled denture base resin treated with Polident; (J) conventional denture base resin treated with Clinsodent; (K) printed denture base resin treated with Clinsodent; (L) milled denture base treated with Clinsodent

The colony count values for milled, printed, and conventionally fabricated samples were done and tabulated using Google Sheets (CFU/mL) (Figure 6).

Statistical analysis

The tabulated data were transferred to IBM SPSS Statistics for Windows, Version 26 (Released 2019; IBM Corp., Armonk, New York) to run statistical analysis at a significance level of α = 0.05. The Kolmogorov-Smirnov tests were employed to evaluate whether the sample followed a normal distribution and exhibited homogeneity. Comparison across all study groups was evaluated using the one-way analysis of variance (ANOVA) test. To demonstrate differences in groups, a post-hoc test was conducted.

## Results

**Figure 7:**
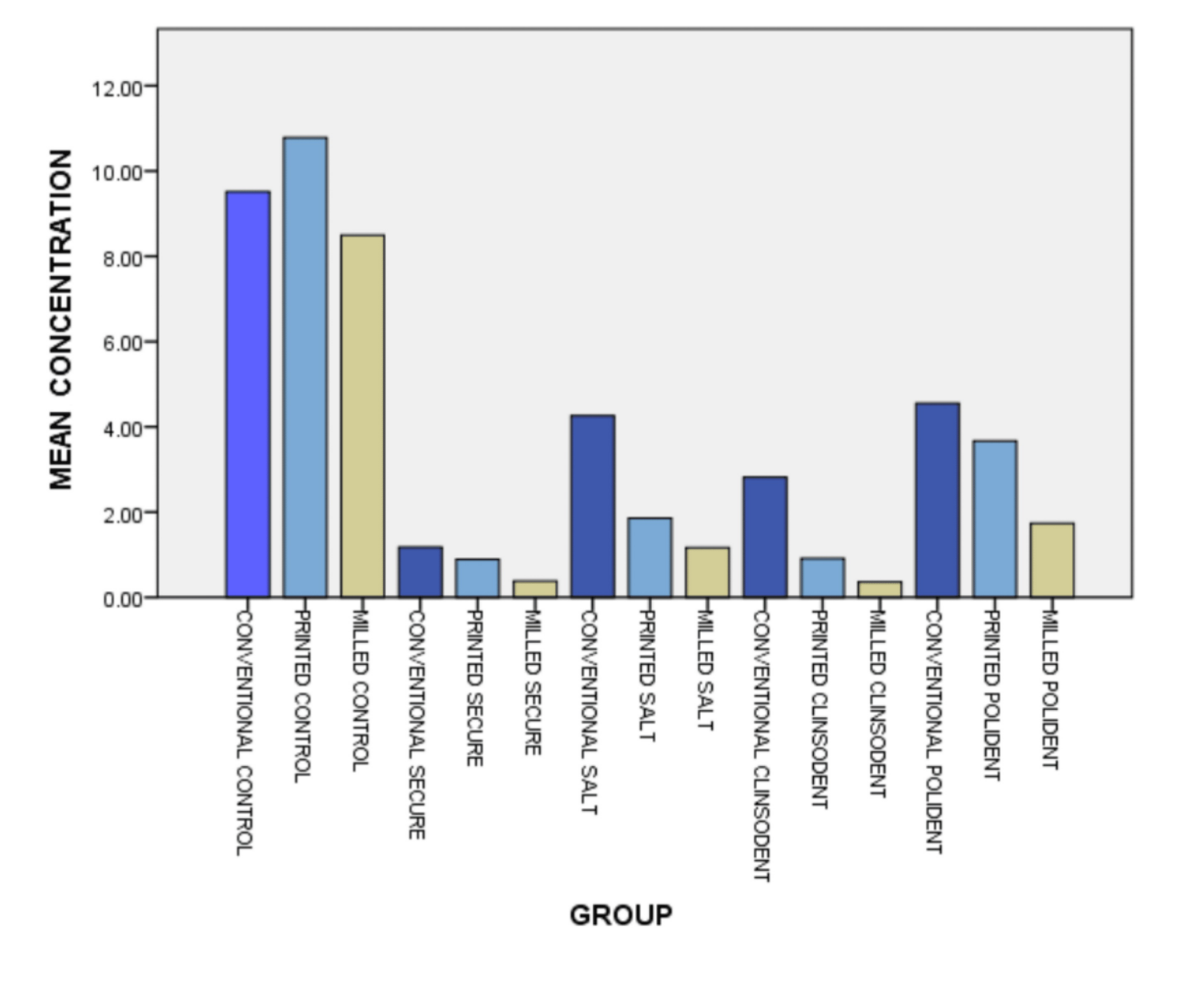
Graph depicting the colony counts before and after treatment of the specimens X-axis: groups; Y-axis: mean concentration (CFU/mL)

The data presented included the reduction in colony count after treatment using the denture cleansers, per the manufacturer's instructions. All the comparisons showed statistically significant differences between the respective groups, as indicated by the p-values (Sig) being 0.000, which is less than the typical significance threshold of 0.05. The 95% confidence intervals for each mean difference do not include zero, further confirming the statistical significance of these differences (Table 3). All specimens showed high numbers of candida colonies after biofilm formation, ranging from 9.2×10^2^ to 11.13×10^2 ^CFU/mL, with milled denture base resins showing the lowest counts. After treatment with cleansers, milled resins continued to show the least candidial colonies. Although lower than their control, the printed and conventional groups showed promising results with Secure, followed by Clinsodent, Polident, and table salt (Figure 7).

**Table 3:** Mean and standard deviation among Groups 1, 2, and 3

Group	Comparison Group	Mean Difference (CFU/mL)	Std. Error	Sig.	95% Confidence Interval	
					Lower bound	Upper bound
Conventional control	Conventional Secure	8.33	0.12832	0.000	7.8936	8.7084
	Conventional salt	5.254	0.12832	0.000	4.8106	5.6974
	Conventional Polident	4.964	0.12832	0.000	4.5206	5.4074
	Conventional Clinsodent	6.699	0.12832	0.000	6.2556	7.1424
Printed control	Printed Secure	9.887	0.12832	0.000	9.4436	10.3304
	Printed salt	8.920	0.12832	0.000	8.4766	9.3634
	Printed Polident	7.114	0.12832	0.000	6.6706	7.5574
	Printed Clinsodent	9.863	0.12832	0.000	9.4196	10.0584
Milled control	Milled Secure	8.114	0.12832	0.000	7.6706	8.5574
	Milled salt	7.329	0.12832	0.000	6.8856	7.7724
	Milled Polident	6.756	0.12832	0.000	6.3126	7.1994
	Milled Clinsodent	8.131	0.12832	0.000	7.6876	8.5744

## Discussion

The primary objective of this study was to compare the fungal colony count and the efficacy of routinely used denture cleansers on newly developed denture base resins. It provides a clear understanding of the biofilm formation of varying materials and the ability of their removal using commercially available denture cleansers as used routinely by denture wearers. The highest efficacy of biofilm removal was seen with Secure denture cleanser on milled denture base resins. Among the three groups, the highest efficacy of all denture cleansers was seen on milled denture base resins. Among denture cleansers, Secure showed the highest effectiveness, followed by Clinsodent and Polident, and the lowest efficiency was observed with table salt.

The poor outcome with Polident could be attributed to its neutral pH, which suggests equal numbers of hydrogen and hydroxyl ions in the solution, indicating a lower degree of hydrolysis [11].

Various studies have reported a direct correlation between the use of denture cleansers and an increase in surface roughness, which in turn leads to more microbial adhesion. Similar results showed the highest surface roughness in conventional resins, followed by printed resins, and the least with CAD/CAM-milled denture base resins [12-15].

Literature has demonstrated the high efficacy of biofilm removal by a hybrid of mechanical cleaning, which includes brushing and using a denture cleanser. Rinsing with plain water showed the lowest efficacy, followed by solely using denture cleansers, followed closely by brushing with water, and the highest efficacy was demonstrated by a hybrid routine [16]. Although recommended by various authors, the use of toothbrushes demonstrated increased surface roughness of denture base resins [17].

Natural, herbal, and ayurvedic at-home remedies have recently become increasingly popular among patients. In a study by Gopal et al., the Red tooth powder showed the highest antibiofilm formation compared to Brindha and Kosali tooth powders [18]. Patients using household items reported using hand soaps, vinegar, and alcohol [19].

The introduction of fillers and nanoparticles in denture base resins has become popular for modifying surface characteristics and, hence, microbial adhesion. In a study by Tsutsumi, the prevalence of *Candida albicans* on the polished surface of PMMA denture base resin can be reduced by the incorporation of at least 5% (w/w) surface reaction-type pre-reacted glass-ionomer filler in the polymer [20]. The incorporation of silver nanoparticles using fungi showed lower adherence of microbes to resins, but the study had a small sample size [21]. The introduction of fillers must be done at the manufacturer level after stringent testing to avoid processing errors and modifications to the surface characteristics of resins, which will be caused by unmonitored quality and quantity of materials.

A total of 73 articles have been reported demonstrating the ill effects of the presence of persulfate in denture cleansers. These included problems ranging from sores to difficulty in breathing and resulted in one fatality. Although making cleansers more viable, the presence of persulfate has been questioned by the Food and Drug Administration (FDA) [22].

Acknowledging the drawbacks of this present study, the biofilm adhesion was considered on newly fabricated specimens, which may alter after use, as surface characteristics are found to change considerably after the aging of acrylic resins [23,24]. Differently polished resins will have varying roughness and, hence, varying biofilm adhesion. Polishing protocols are subjective and vary based on the operator, burs, and materials used. The availability of burs and polishing pastes depends on location and financial capabilities.

## Conclusions

Within the limitations of this study, the highest candida colony count was demonstrated by the conventional method, followed by rapid prototyping, while the milled denture base resins had the lowest count. Following treatment with denture cleansers, Secure demonstrated almost complete eradication of colonies, deeming it the most effective option. Salt exhibited the lowest efficiency, followed closely by Polident and Clinsodent, and the most efficacious was Secure denture cleanser.
